# Morph specific foraging behavior by a polymorphic raptor under variable light conditions

**DOI:** 10.1038/s41598-017-07829-x

**Published:** 2017-08-22

**Authors:** Gareth J Tate, Arjun Amar

**Affiliations:** FitzPatrick Institute of African Ornithology, DST-NRF Centre of Excellence, University of Cape Town, ZA-7701 Rondebosch, South Africa

## Abstract

Colour polymorphism may be maintained within a population by disruptive-selection. One hypothesis proposes that different morphs are adapted to different ambient light conditions, with lighter morphs having a selective advantage in bright conditions and darker morphs having advantages in darker conditions. The mechanism for this advantage is proposed to be through enhanced crypsis via background-matching. We explore this hypothesis in a polymorphic raptor, the black sparrowhawk *Accipiter melanoleucus*, which exhibits a discrete dark and white-morph. We use GPS-tracking data to contrast the foraging behaviour and habitat selection of morphs. As predicted, we found that light-levels influenced foraging behaviour in different ways for morphs: Dark-morphs showed a decrease in foraging with increasing light-levels; whereas no relationship was found for white-morphs. Furthermore, we found differential-degrees of habitat selection, with dark-morphs selecting more enclosed habitats compared to white-morphs. This suggests that different morphs may be better adapted to foraging under different light-conditions, potentially playing a role in maintaining colour polymorphism in this species. Our results may also help explain why dark-morphs predominate in this study region, which experiences high rainfall and lower light-levels during the breeding-period. This study suggests that avian morphs may allocate/partition foraging activity by weather conditions/habitat, which maximise their concealment from prey.

## Introduction

Colouration has been widely recognised to play an important role in a variety of ecological processes, from camouflage (crypsis)^1^ to intraspecific communication^2^ and mate choice^3^, all of which are likely to be under considerable selective pressure from both natural and sexual selection^4^. Thus, the colour of an individual animal, a population, or a species, is thought to be shaped by complex evolutionary processes that drive and maintain phenotypic variation and genetic diversity in nature^5^.

The plumage colours and patterns that bird species display have fascinated evolutionary biologists for decades, although general explanations for them still often remain elusive^6^. For some bird species, colouration and patterning in their plumage is likely to play a vital role in camouflage and crypsis via background matching^1, 4^. According to this principle, the more similar to the visual background the colours and geometry of patterns of an individual are, the more concealed it should be^2^. This can be fundamental either for prey to avoid detection by predators^1, 7^ or equally so, for predators to avoid detection by their prey^8, 9^. For example, in nightjars (Caprimulgiformes), the colour and plumage pattern of incubating females closely matches their surrounds, reducing their detection from visual predators, improving their overall chances of survival^1^.

Given the likely adaptive value of colour^10^, it is therefore logical to assume the same processes may be operating on colour polymorphic species, where multiple genetically based colour morphs occur within the same age and sex class of a breeding population^11^. Consequently, evolutionary biologists have frequently used colour polymorphic species as model systems for the study of important evolutionary processes such as natural and sexual selection^12^ and specifically, to test how colour variants (i.e. multiple morphs) are maintained in species in the face of natural selection and genetic drift^13–15^. In theory, the principles of natural selection predict that the fittest morph should be selected and that morphs with lower fitness should be eliminated from a population^15^. Therefore, it has been proposed that for multiple morphs to coexist, and for polymorphism to be maintained within a species, morphs must have equal fitness over time^5^; whereby, a selective trade-off exists between alternative morphs, both enjoying some advantages, but frequently also incurring some costs^16, 17^.

Colour polymorphism occurs in 3.5% of bird species globally^18^, although there is considerable variation between different groups. It is particularly common amongst raptors (owls and hawks), where 30% are polymorphic^18^. Numerous studies have suggested that different morphs may represent alternative strategies adapted to certain prevailing environmental conditions^19^. Disruptive selection, in which selection favours contrasting phenotypes under different environmental conditions, has been invoked as one of the main mechanisms maintaining colour polymorphism^18^.

As ambient light condition strongly influences the level of crypsis in certain colours^20, 21^, and also affects the ability for prey to visually detect predators, or for predators to detect prey^7^, light could play an important role in driving disruptive selection in polymorphic species. Indeed, based on their review and analysis, Galeotti *et al*. (2003) concluded that colour polymorphism in birds probably evolved under selective pressures linked to individual detectability under variable light conditions and argued that it is most likely maintained by disruptive selection. In predator-prey systems, dark and light plumage may therefore be beneficial under alternating light conditions. For example, darker morphs may be less detectable in low light conditions (i.e. on cloudier days, during dusk/dawn, or in closed habitats), and in these conditions darker predators may accrue a foraging advantage via background matching^8, 22^. Conversely, lighter morphs may be less detectable in brighter conditions and may forage more successfully as a result under these conditions (i.e. on sunny days, in the middle of the day, or in open habitats)^18^.

Galeotti *et al*.’s (2003) hypothesis has recently received some empirical support; Tate *et al*. (2016) found that prey delivery rates for black sparrowhawks (*Accipiter melanoleucus*) were significantly higher for dark morph individuals during low light conditions, whereas the opposite relationship was found for white morphs, which appeared to have an advantage in brighter conditions. These findings suggest that light conditions interact with morph colour to improve foraging success and supports the hypothesis that the two morphs (Supplementary material Fig. 1) may be better adapted to foraging under different light conditions (see also ref. 23). Within South Africa, black sparrowhawks display clinal variation in morph ratios, with the frequency of dark morphs declining from >75% in the southwest to <20% in the northeast of the country^24, 25^. This variation is most closely correlated with ambient light levels during the winter period, with higher proportions of dark morphs being associated with darker winter conditions^24^ and correspondingly with higher winter rainfall^25^. Within our study population, on the newly colonised Cape Peninsula^26^, where rainfall during the winter breeding period is particularly high^27^, the dark morph phase predominates, with >75% dark morphs in the breeding population and these morph ratios remaining stable for over a decade^24, 25, 28^.

**Figure 1:**
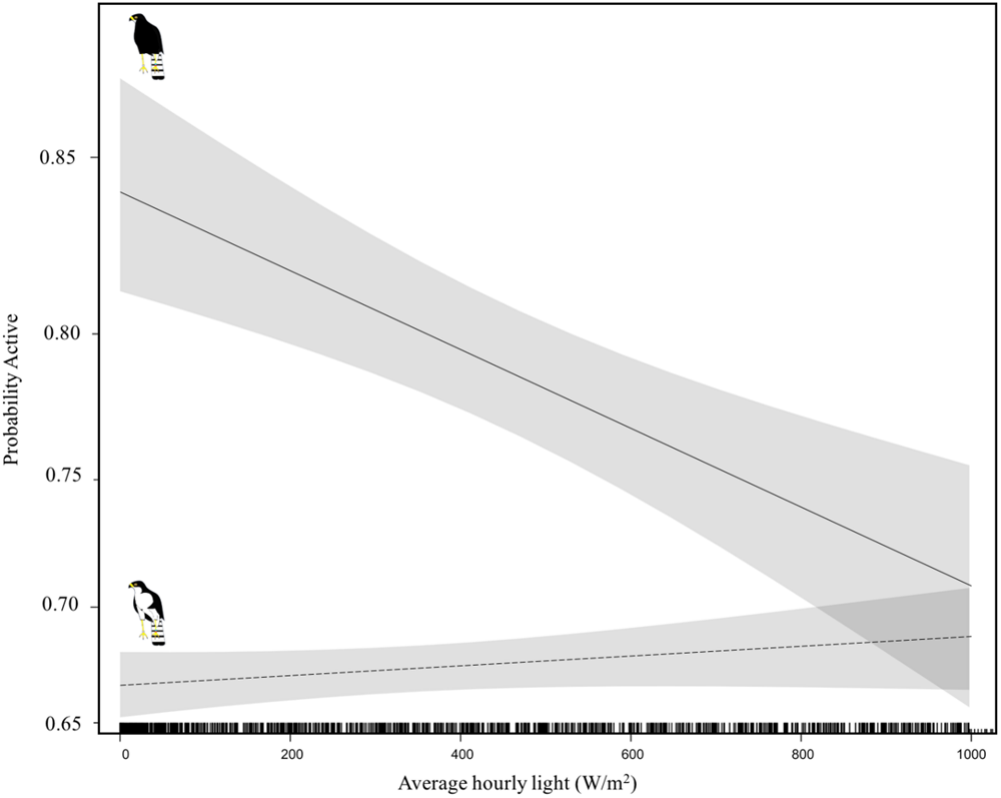
Foraging activity (measured as presence or absence from the core nesting area (10% kernel estimate) of black sparrowhawks on the Cape Peninsula across the ambient light spectrum with 95% confidence intervals. During the breeding season, we found a significant interaction between light level (W/m^2^) and morph (χ^2^_1_ = 18.2, P = 0.01). Controlling for breeding stage, for white morph birds, foraging activity increased slightly as conditions became brighter (P = 0.8), whereas activity levels for dark morphs declined substantially and significantly as light levels increased (P < 0.001). Examining this relationship in more detail, dark morphs remain more active below around 800 W/m^2^, in conditions brighter than this there was little difference in activity levels away from the nest between the two morphs, as can be seen from the overlapping confidence limits. “Morph images” by Ann Koeslag.

In this paper, we further explore the hypothesis tested by Tate *et al*. (2016) using the same study system. In their study, Tate *et al*. (2016) examined provisioning rates as a surrogate for foraging success in relation to ambient light levels. In this study, we explore the issue more directly using GPS tagged male black sparrowhawks, to investigate whether activity and foraging effort differs between the morphs depending on light conditions. Black sparrowhawks typically attack their avian prey from above via short ambush flights^29^ and appear to accrue foraging benefits/advantages in ambient light conditions (habitat/time of day or rainy vs cloudy days) that best conceal them from their prey against the background^24^. If foraging success is influenced by light levels differently for the two morphs in the manner expected, we predict that dark morphs will forage more during lower light conditions, when they would be expected to be most successful, whereas white morph birds will show the opposite relationship. Furthermore, we also test whether habitat selection varies between the morphs. Here we predict that dark morph birds will show a greater preference for more enclosed habitats (which should represent darker habitats) than white morph birds, which might show a greater preference for more open habitats.

## Results

### Foraging activity and effort in relation to light levels

During the active breeding season (April–October, with peaks in May and August), we found that overall foraging activity (i.e. fixes away from core nesting territories) was greater in dark morphs (mean ± SE = 0.84 ± 0.17, range = 0.82–0.89) than white morphs (mean ± SE = 0.65 ± 0.16, range = 0.59-0.70) (χ^2^_1_ = 16.5, P < 0.001). There were also significant differences in foraging activity across the four different breeding stages (χ^2^_1_ = 55.60, P < 0.001; supplementary material Fig. 2), with birds spending greater periods of time away from the nests during prelay, nestling and fledgling stages compared to the incubation period. After controlling for breeding stage, there was a significant interaction between morph and light levels (χ^2^_1_ = 18.2, P = 0.01); for white morph birds, foraging activity was not influenced by light conditions (χ^2^_1_ = 8.7, P = 0.8; Fig. 1), whereas activity levels for dark morphs declined substantially and significantly as light levels increased (χ^2^_1_ = 4.8, P < 0.001; Fig. 1). Examining this relationship in more detail, below around 800 W/m^2^ dark morphs remain more active than white morphs, whereas in conditions brighter than this there was little difference in foraging activity between the two morphs, as can be seen from the overlapping confidence limits (Fig. 1). Our model including light levels and morph better explained variation in foraging activity than the other models which substituted other weather variables (hourly rainfall or temperature) or time for day (and its quadratic term) (Table 1). A modified analysis using a similar, subsampled number of fixes for dark and white morphs, also showed a significant interaction between light level (W/m^2^) and morph (χ^2^_1_ = 36.37, P < 0.001). Controlling for breeding stage, for white morph birds, foraging activity decreased slightly as conditions became brighter (P = 0.7), whereas activity levels for dark morphs declined substantially and significantly as light levels increased (P < 0.001)

**Figure 2:**
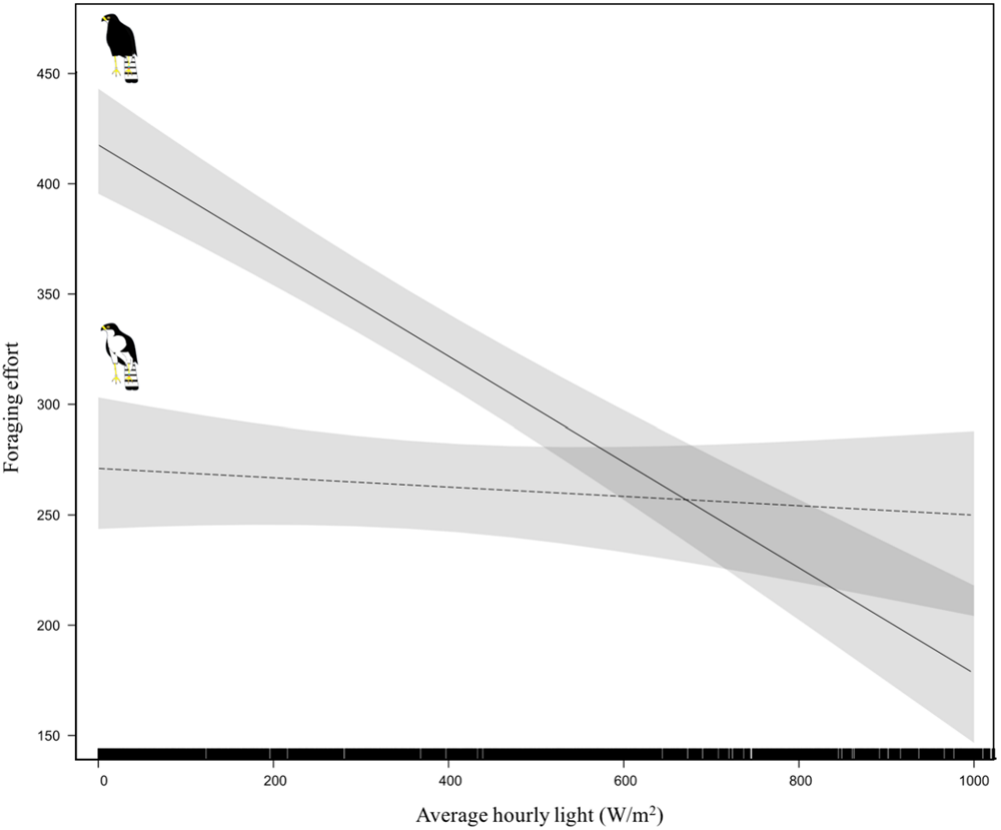
Foraging effort, measured as the average hourly distance travelled (meters) by individuals outside of the core nesting area (10% kernel estimate) with 95% confidence intervals. We detected a significant interaction between light levels and morph, with foraging effort decreasing as conditions became brighter for dark morphs. The same trend was apparent for white morphs but there was no significant difference in foraging effort across the light spectrum.

**Table 1:** Models explaining variation in foraging activity (presence or absence from the core nesting area (10% kernel estimate)). Model selection based on Akaike Information Criterion scores (AIC). Results show the top ranked model included light level (measured as solar radiation (W/m^2^)) and held 100% of the weight. The analysis revealed that light level and its interaction with morph was the better explanatory variable when compared with either temperature, rainfall or time of day. AICc weight (*W*_*i*_). Rain = average hourly rainfall, Temperature = average hourly temperature and Time = time (and its quadratic term). K denotes the number of parameters (K), change in AIC relative to the highest ranked model (ΔAIC), AIC weight (*W*_*i*_). Morph = M, Light = L, Breeding stage = BS, Time = Ti, Temperature = Te, Rain = R.

**Model**	**K**	**AIC**	**∆AIC**	***W***_***i***_	**Cum. Wt**
M + L + BS + M* L	8	24653.02	0.00	1	1
M + (Ti + Ti^2^) + BS + M*(Ti + Ti^2^)	10	24824.1	171.08	0	1
M + Te + BS + M*Te	8	24843.15	190.12	0	1
M + R + BS + M*R	8	24978.04	325.02	0	1

We found a very similar relationship for our secondary foraging measure – foraging effort, which was the average hourly distance travelled. For this term no overall differences were noted between the morphs (χ^2^_1_ = 1.027, P = 0.3). Foraging effort did, however differ between breeding stages (χ^2^_1_ = 32.40, P = 0.004), with greatest foraging effort, and distances being covered, during incubation and nestling stages and lowest during prelay and fledgling stages (Supplementary material Fig. 2). After controlling for breeding stage, we again found a significant interaction between light levels and morph (χ^2^_1_ = 28.66, P = 0.002; Fig. 2). The interaction was similar in nature to our previous result for foraging activity, with foraging effort remaining similar across light levels for white morphs (P = 0.9), but decreasing significantly as conditions became brighter for dark morphs (P < 0.001). Again we found that our models including light levels and morph better explained variation in foraging effort than models including the other weather terms or time (and its quadratic term) (Table 2).

**Table 2:** Models explaining variation in foraging effort (Average hourly distance travelled by individuals outside of the core nesting area (10% kernel estimate)). Model selection based on corrected Akaike Information Criterion scores (AIC). Light level measured as average hourly solar radiation (W/m^2^). Rain = average hourly rainfall, Temperature = average hourly temperature and Time = time (and its quadratic term). K denotes the number of parameters (K), change in AIC relative to the highest ranked model (ΔAIC), AIC weight (*W*_*i*_). Results show the top ranked model (with ΔAIC < 2, shown in bold) included the term light level and its interaction with morph and held 100% of the weight. Morph = M, Light = L, Breeding stage = BS, Time = Ti, Temperature = Te, Rain = R.

**Model**	**K**	**AIC**	**∆AIC**	***W***_***i***_	**Cum. Wt**
M + L + BS + M* L	9	131271.6	0.00	1	1
M + Te + BS + M*Te	9	131442.1	170.46	0	1
M + R + BS + M*R	9	131573.1	301.46	0	1
M + (Ti + Ti^2^) + BS + M*(Ti + Ti^2^)	11	132306.1	1034.53	0	1

### Morph habitat preference

**Figure 3:**
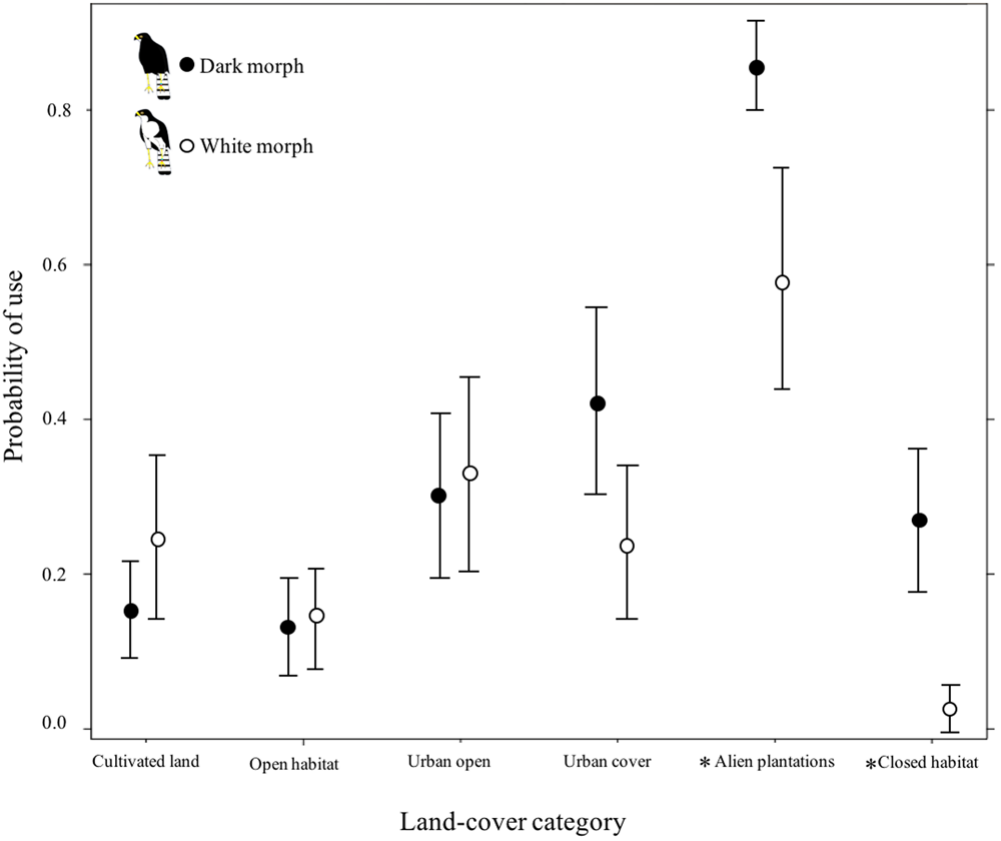
Morph specific habitat selection on the Cape Peninsula. The two morphs differed in their level of habitat selection; with no obvious difference in selection between the morphs ﻿fo﻿r the more open habitats of ‘urban open’ (Lsmeans pairwise test, P = 0.9), ‘cultivated land’ (Lsmeans pairwise test, P = 0.8) and ‘open habitat’ (Lsmeans pairwise test, P = 1) than dark morphs. Conversely, dark morphs showed significantly greater selection for the more closed ‘alien plantations’ (Lsmeans pairwise test, P < 0.001) and ‘closed habitat’ (Lsmeans pairwise test, P < 0.001) than white morphs. Morphs had a similar selection for the habitat ‘urban cover’ (Lsmeans pairwise test, P = 0.2). Bars indicate one standard error.

**Figure 4:**
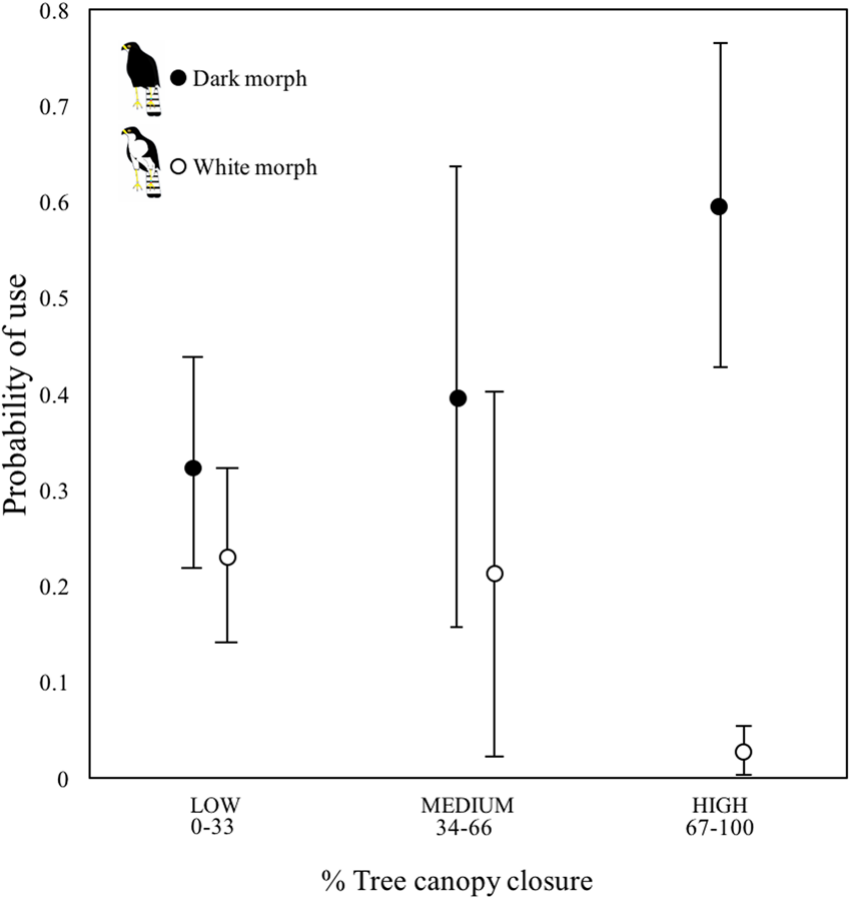
Probability of use ( ± 1﻿ SE) of areas with different canopy cover levels (percentage of canopy closure) by male black sparrowhawk morphs on the Cape Peninsula. Dark morph males had a significantly higher probability of using high tree cover (67–100% canopy cover) compared to white morphs (χ^2^_1_ = 11.71, P < 0.001)). No significant differences in selection were found for low (0-33%) (χ^2^_1_ = 3.13, P = 0.07) or medium (34-66%) canopy cover (χ^2^_1_ = 0.27, P = 0.6).

Across our six habitat types, we detected differences in the degree of habitat selection between white and dark morph black sparrowhawks, that were close to significant (χ^2^_1_ = 3.10, P = 0.05; Fig. 3). Our post-hoc analysis, however, revealed significant differences between the two morphs in their level of habitat selection for the two most enclosed habitat types; with dark morphs showing significantly greater selection for the more enclosed ‘alien plantations’ (Lsmeans pairwise test, P < 0.001) and ‘closed habitat’ (Lsmeans pairwise test, P < 0.001) than white morphs. Lastly, exploring habitat use in relation to the percentage of tree canopy cover/closure between the morphs, we found a highly significant interaction between male morph type and the percentage of tree cover (χ^2^_1_ = 87.33, P < 0.001). Analysing this further using a moving window analysis, we found that dark morphs displayed a significantly higher probability of using habitats with denser tree canopy cover (67-100% canopy cover) compared to white morphs (χ^2^_1_ = 11.71, P < 0.001) (Fig. 4). However, no significant differences in selection were found for low (0-33%) (χ^2^_1_ = 3.13, P = 0.07) or medium (34–66%) canopy cover (χ^2^_1_ = 0.27, P = 0.6).

## Discussion

Our tracking data enabled us to compare foraging activity, foraging effort and habitat selection between the two morphs in our study population of black sparrowhawks. Although our sample size was small, with only 3 birds of each morph tracked, our results did provide support for the hypothesis that the two morphs are adapted for foraging in different light environments. We found support for both our predictions; dark morphs displayed greater foraging activity and effort in lower light conditions and showed a stronger degree of selection for enclosed habitats with greater vegetation cover. In contrast, white morphs displayed no significant relationships in their foraging activity and effort in relation to light conditions; however they did show weaker selection for more enclosed habitat types than dark morphs. The fact that we found support for these predictions with our relatively small sample size suggests the nature of these relationships are pervasive among individuals of the two morphs. However, because sample sizes were small we cannot, of course, completely rule out the notion that these results simply arose by chance, with the three dark birds displaying this relationship with light levels that were due to their inherent nature rather than their morph colour, or as a result of different sampling windows and collection times. Only a larger sample of birds could demonstrate this more effectively. Our adjusted analysis using random subsampled fixes from our dark birds to have a similar number of fixes as white birds produced nearly identical results, suggesting that differing sample sizes between the morphs is unlikely the main driver for the results.

In this study, our assumptions are that morphs would invest most time into foraging activities during the light conditions or within the habitats, that will maximize their foraging success, as would be expected under optimal foraging theory^30^. Our results are generally consistent with the findings from Tate *et al*. (2016), which found that prey provisioning rates to nests were associated with light levels differently between the morphs in the same study population. These results, from provisioning rates to nests, are unlikely to have been confounded by differential prey sizes for two reasons; i) because within our study area >80% of prey items consist of 4 similar sized dove species (*Streptopelia semitorquata, Columba livia, Columba guinea, Streptopelia capicola)* ^31^ and ii) we have found no significant differences in diet composition between morphs^24^. Thus, from our results it appears that dark morphs make trips away from the nest site more often, and hunt with greater effort under light conditions where they may benefit from improved crypsis. As light levels increase, foraging presumably becomes less profitable for this morph and so they become less active and thus cover smaller distances on these trips. The results from this study, in combination with those from Tate *et al*. (2016), may provide further information on how light levels may influence foraging success of the two morphs. For example, white morphs appear to spend the same amount of effort foraging irrespective of the light levels; however, they provide more prey during brighter conditions^24^. The most likely explanation for this is that this morph has higher capture rates during brighter conditions, presumably due to their improved crypsis^24^. However, in contrast, the results do not necessarily support the conclusions of Tate *et al*. (2016) for dark morphs. For dark morphs, both foraging effort and provision rates were influenced in a similar manner by light conditions, with both measures increasing by around 50% over the light spectrum. This result is therefore suggestive that prey capture rate for dark morphs does not vary in relation to light levels (*cf*. white morphs), as was previously suspected, but rather that it is proportional to the amount of time spent hunting. Unfortunately, it is very difficult to measure hunting success directly. However, with the novel use of accelerometers in tags, this may soon be possible and could usefully be applied to understand the relationships between hunting and light levels in these different morphs^32^.

In our analysis, we found that light conditions in the study area were the most important predictor of morph foraging activity and effort, which has important implications for understanding how polymorphism is maintained in our study system. The ambient light intensity within an environment during the course of the day, between habitats or in different weather conditions (i.e. cloud cover) can vary considerably^4, 21, 33^ and is likely to be a strong selective agent^18, 34^. In many predator-prey systems, individuals may therefore gain improved crypsis, and thus enjoy selective advantages such as enhanced hunting success, under certain ambient light conditions^18^.

Background matching is strongly enhanced by the amount of ambient light in an environment^2, 21^, whereby darker colours are harder to visually detect in low light conditions^8^, and lighter colours are more cryptic in brighter conditions^9, 21^. Ambient light is significantly different between open and closed habitats, with shading from vegetation strongly reducing light levels^21^ (i.e. in the order of 300 billion fold; Martin 1990). Thus, the interplay of background colour, coupled with light condition, may have substantial influence on an individual’s detectability or level of crypsis (see supplementary material Fig. 4). Background matching with surrounding habitat, and light condition below vegetation canopy, may therefore represent an important factor in the maintenance of colour morphs in predator-prey systems, and may explain why polymorphism is most common in species living in both open and closed habitats^18^ and across spatially variable environments or backgrounds^4, 34^.

Several studies have documented differential habitat selection across morphs, often within the same population^35^. This habitat preference has frequently been linked with foraging benefits accrued for different morph types in certain habitats^36^. For example, in the red-tailed hawk (*Buteo jamaicensis*), light morph birds were observed to occupy more open perch sites when hunting, compared to dark morphs^22^. Rohwer (1990) found that the two morphs of the Pacific reef heron (*Egretta sacra*) used different hunting techniques and utilized different habitat types within the same locality, with dark morphs foraging almost exclusively under dense canopy cover, while light morphs foraged more often in brighter, open habitats. A study on the tawny owl (*Strix aluco*) also provides data supporting that dark (rufous) morph owls inhabit more wooded and dense-covered territories than light (grey) morphs^37^. The observed morph habitat preferences found in our study also supports an adaptive morph-habitat association. Our findings further reinforce the conclusions from other studies^36, 37^ and supports the idea that black sparrowhawk morphs forage during conditions, and within habitats (related to the light conditions in a specific habitat), that are likely to enhance their crypsis from their avian prey.

Numerous studies have shown that body colouration may be associated with environmental factors that are not related to crypsis, hunting or predation. These include correlated physiological effects which may lead to geographically related advantages of different morphs, such as thermoregulation^4^, optimal heat or water balance, or improved protection against UV-radiation and feather abrasion^38^. For example, in some polymorphic reptiles, dorsal body colour was found to be strongly correlated with the thermal environment, thus, serving a thermoregulatory purpose^39^. In the bananquit (*Coereba flaveola*), dark morphs were found to prefer shaded, wetter habitats over the brighter yellow morphs in the absence of predation and thus have no purpose for enhanced crypsis^35^. Therefore, activity and behaviour in some species may not be due to variable capture success or crypsis under varying light conditions, but rather related to intrinsic behavioural differences between morphs in their coping strategies under variable light conditions.

Although these physiological correlates are, in theory, credible explanations for the presence of morphs in some environments, our results strongly suggest that light levels explained variation in foraging activity and effort in the black sparrowhawk better than other weather conditions or the time of day. Our findings are further reinforced by the fact that black sparrowhawk morphs display ventral plumage polymorphism, a side most obvious to their avian prey below. This plumage pattern conforms to the classic example of countershading proposed under Thayer’s law^10, 40^ and is a common pattern seen in predators of many taxa, particularly those which attack their prey from above (see Götmark’s (1987) experiment dying the underparts of black-headed gulls, *Larus ridibundus*). This fits with the mode of attack of black sparrowhawks, which mainly attack via short ambush flights when their bird prey is on the ground^29^. Ventral colouration is therefore considered to have an important role in decreasing the conspicuousness of predators against their background^9, 40, 41^.

Our findings also offer an explanation for the numerical dominance of dark morphs on the Cape Peninsula, where >75% of birds are dark morphs^25^. The recent range expansion in black sparrowhawks has brought the species into contact with a novel climatic regime^27^, particularly during the species winter breeding season. Throughout South Africa, the species distribution is now characterized by two contrasting climatic windows, with dry winters in their historical range in the north and east, and wet winters in the recently colonized Western Cape region^42^. The Cape Peninsula receives particularly high levels of rainfall as a result of its exposure to southern oceanic fronts and the orographic effects of Table Mountain^27^. Therefore, local light conditions are particularly low during the wet breeding season and, under these conditions, dark morphs have been shown to have a potential advantage over white morphs via improved foraging success^24^. These local breeding conditions may therefore be important in maintaining a higher than expected proportion of dark morphs in our study population of black sparrowhawks. For example, Tate *et al*. (2016) showed that for the majority of the winter breeding period, light levels are below that where white and dark morphs show similar foraging behaviours. This is further reinforced by recent findings by^23^, which revealed that recruitment of chicks strongly depended on their father morph in associated with the timing of breeding. Significantly higher recruitment rates were reported for nests with dark morph males breeding earlier on in the season (when light conditions are generally lower), whereas higher recruitment rates were documented in nests with white morph males breeding later in the season (when conditions became brighter).

Interestingly, although winter breeding conditions (i.e. the predominantly low light conditions) on the Peninsula appear to favour dark morphs in terms of their hunting success, this is not being translated into overall advantages in other fitness related traits, such as survival and breeding performance. In fact, recent work conducted on the same study population of black sparrowhawks has revealed that survival and productivity does not differ between morphs at the individual level, however, the study did show that the morph combination of breeding adult pairs influenced productivity significantly, with mixed-morph pairs producing more offspring (around a quarter more) per year than pairs consisting of the same morph^43^; although double brooding has been recorded in the study population^44^, black sparrowhawks tend to only have one brood per season. The authors propose that the higher productivity of mixed-pairs may be the result of the complementary nature of care provided by the different morphs, with differential foraging success between black sparrowhawk morphs under varying light conditions^24^ allowing mixed-pairs to expand their foraging niche (i.e. across the full light spectrum (low and bright light conditions)). The results from this study also suggest this may also extend, to some degree, to different habitats types and specifically, vegetation cover. Thus, mixed pairs will be able to exploit a wider range of habitat types, than like-pairs. Subsequently, emergent pair-level properties may play an important role in promoting and maintaining polymorphism in the species.

In this study, we have assumed that when birds are outside of their core nesting territories, they are actively foraging, however, we recognise that they may have also been engaging other activities such as territorial behaviour, or avoiding begging females and chicks (although the inclusion of foraging effort in our separate analysis should account to a degree for this potential bias). Numerous studies on polymorphic bird species have documented differences in aggression across morphs^45^, with melanistic morphs frequently displaying higher levels of aggression, and lower sensitivities to stress, compared to lighter morphs^13, 45^. Therefore, rather than engaging in foraging activities, dark morph black sparrowhawks may be making more regular trips away from their nest sites as they are defending their breeding territories more actively and aggressively, and therefore appear more active than white morphs. Extra pair copulations (EPC) has also been observed in our study population^42^, and it may be that dark morphs spend more time away from their nests actively pursuing and securing additional mates compared to white morphs, a trait that has been witnessed in other polymorphic bird species^46^. Whilst this might explain the higher overall levels of activity seen for the dark morphs, it does not explain the contrasting relationships in activity in relation to light levels seen for the different morphs in this study.

The current study represents the first attempt to explore whether foraging behaviour by different morph types is influenced differently by ambient light conditions. Our findings suggest that the two colour morphs in the black sparrowhawk have differential foraging activity and foraging effort across variable light conditions. Additionally, we found morph differences in habitat and cover preference-likely related to the light conditions provided by these habitats. This appears to be linked with the conditions under which they have improved crypsis and thus optimum foraging success^24^. Our study suggests that a significant foraging advantage under local light and habitat conditions may be important in maintaining a higher than expected proportion of dark morphs in the Cape Peninsula population of black sparrowhawks. As breeding light conditions throughout South Africa are likely to be highly variable as a result of the different climatic regimes that occur across the black sparrowhawks distribution, our study provides important supporting evidence for the role of breeding season light levels in spatial structuring (i.e. clinal variation^25^) of morphs across the landscape of South Africa, as established by Tate *et al*. (2016). Interestingly, many *Accipiters* share a similar type of colour polymorphism to that shown by the black sparrowhawk. It would therefore be particularly interesting to repeat these analyses for other polymorphic *Accipiter* species, to see whether similar relationships hold and therefore whether generalizations can be drawn across the polymorphic *Accipiters*.

## Methods

We examined morph specific foraging behaviour and habitat use in a newly colonised black sparrowhawk population on the Cape Peninsula (34°00′S, 18°26′E) Western Cape, South Africa. The study area encompasses a variety of natural and human modified (urbanised) environments which include the slopes of the Cape Peninsula Mountain chain and adjacent sand flats, with altitude ranges from sea level to about 400 m. The region features a mosaic of urban gardens and green belts, golf courses, schools, small pockets of indigenous afromontane forest, botanical gardens, fynbos shrubland and alien pine (*Pinus* spp.) and eucalyptus (*Eucalyptus* spp.) plantations-particularly along the eastern slopes^44^.

### Tracking data

Six adult male black sparrowhawks (3 white and 3 dark morphs) were trapped on active territories, using a *bal-chaltri* trap baited with live white pigeons (*Columba livia*)^47^ and fitted with GPS loggers using a back pack harness made of 7mm teflon ribbon (Bally Ribbon Mills, Bally, PA, USA). All information on the tracking data obtained for each individual is provided in Table 3. We focused on males as they undertake the majority of the provisioning during the breeding season, while females incubate and brood chicks^48^. Tags were solar powered EP3.5 ‘Harier’ loggers manufactured by ECOTONE (Sopot, Poland). Our sample size was limited by the fact that male black sparrowhawks are difficult to trap, are present at the nest less frequently than females^43^ and by the expensive costs of the tags themselves (~US $1350/tag). Additionally white morph males are particularly rare in our population^29^. All loggers were set to record GPS fixes (accurate to within 1-15 m) every fifteen minutes on a daily cycle from 4am to 9 pm (SAST). Fixes recorded on the loggers were downloaded via Very High Frequency (VHF) transmission once a month using a portable base station and directional antenna. Downloads could occur when the base station was within c. 4 km of the tagged bird.

**Table 3:** Summary of our main GLMM analyses examining morph specific foraging activity and foraging effort across the ambient light spectrum, as well as level of habitat selection, using six habitat categories (HAB) and percentage tree canopy closure (TREE). Explanatory variables were M = morph type (dark or white) L = light level (W/m^2^) and breeding stage (BS). Additional analyses (in parentheses) were also undertaken for foraging activity and effort where light level was replaced with either temperature (Te), rainfall (R) or time (and time^2^) (Ti).

**Analysis**	**Family**	**Random term**	**Response variables**	**Explanatory variables**
Foraging activity	Binomial	Individual	binary response variable: #fixes actively foraging / #fixes not foraging	M + L + BS + M*L (Te,R,Ti)
Foraging effort	Gaussian	Individual	Average hourly distance travelled	M + L + BS + M*L (Te,R,Ti)
Use by Habitat	Binomial	Individual	binary response variable: presences / pseudo-absences	M + HAB + M*HAB
Use by Canopy cover	Binomial	Individual	binary response variable: presences / pseudo-absences	M + TREE + M*TREE

### Ethics

All research was approved by the University of Cape Town’s Science Faculty Animal Ethics Committee (Permit number 2012/V37/AA). The methods were carried out in accordance with the Ethics Committee’s relevant guidelines and regulations; GPS loggers weighed around 14 grams, which is 2.6% of the average male weight (~540 g; Hockey *et al*. 2005), which falls within the ethically approved weight for loggers (i.e. <5% of the species total weight^49^).

### Weather data

For all monitored individuals, we matched each hour of GPS data with the average ambient light that fell during the hour, from a nearby South African Environmental Observation Network (SAEON) weather station (33.952° S, 18.459° E), positioned in an open field, on average 12.2 ± 21 km from the territories of our tagged birds. For ambient light, we used average hourly irradiance i.e. solar radiation in W/m^2^, hereafter referred to as light level^24^. We also gathered information from the same weather station on hourly rainfall (mm) and temperature (°C) which we also linked to the tracking information.

### Habitat data

To examine whether there were any differences in habitat preference between the morphs, a Geographic Information System (GIS) was used to explore the underlying habitat for each GPS fix. For habitat, we collated data for the Cape Peninsula using a national landcover dataset for South Africa, developed by the South African National Biodiversity Institute in 2013/14 (SANBI; http://bgis.sanbi.org/DEA_Landcover/project.asp). The national landcover dataset is based on 30 × 30 meter raster cells and has been derived from multi-seasonal Landsat 8 imagery, using operationally proven, semi-automated modelling procedures developed specifically for the creation of this dataset, based on repeatable and standardised modelling routines^50^. The dataset has been created by GEOTERRAIMAGE (GTI)^50^ and has a total of 72 landcover classes. However, for our habitat preference models, we grouped the SANBI landcover data into the following six categories, ‘cultivated land, open, urban open, urban cover, plantations and closed vegetation’.

For a separate analysis focusing on the potential differences in morph-specific tree cover preference, we collected data on the percentage of tree canopy cover on the Cape Peninsula, using the Hansen global tree canopy cover layer^51^. This data set displays tree canopy cover, defined as canopy closure for all vegetation over five meters in height, over all global landcover at 30 × 30 m resolution, encoded as a percentage per output grid cell, in the range 0–100%.

The habitat in some territories has been dynamic over the duration of this study, with some clearing of alien plantations since 2012, coinciding with when our first individual was GPS tagged. Therefore to create an up-to-date dynamic layer of landcover and tree cover on the Cape Peninsula, we projected our habitat layers onto a high resolution satellite areal image of the study area (provided by the ESRI World imagery base map; http://server.arcgisonline.com/arcgis/rest/services/ESRI_Imagery_World_2D/), and manually updated areas where and when trees had been clear-felled. This was processed in the program QGIS^52^.

We projected all GPS fixes onto the high resolution landcover and tree canopy cover layers for the study area. We used QGIS to process this information and to extract tree canopy cover data for each GPS fix, and we used the statistical software R, version 3.2.2 ©^53^ with the package “raster” to extract data from the SANBI landcover dataset.

### Statistical analysis

For all spatial analyses, GPS fixes were projected to the UTM coordinate system (WGS 1984 UTM Zone 35 S)^54^ and we only used GPS data from actively breeding individuals i.e. during their prelay, incubation, nestling and fledgling stages (Table 4). For breeding data, territories were monitored approximately monthly for signs of occupation and then more frequently (approximately every 2 weeks) once breeding was detected. Black sparrowhawks are territorial tree nesters, usually nesting in large *Eucalyptus* and *Pinus* tree species^29^ and establish territories well before eggs are laid. In dense urban populations like our study population, active nests are typically >1 km apart. The prelay period was classified as the stages leading up to breeding; when birds were courting, mating, nest building and females started going into egg-laying lethargy^55^, a stage where we observed males start to provision their partners with the majority of their food. The incubation period included egg laying (usually clutches of 1–3) until chicks hatched, with incubation lasting between 37–38 days^44^. The nestling period is 40–47 days and the post-fledging period, where chicks leave the nest, however, are still dependent on their parents, can exceed 80 days^44^. Duration of breeding periods differed in length between different pairs, particularly during prelay stages when males had to secure territories and provision their mates. Locational fixes were sufficiently regular and recorded in enough volume to be assumed to approximate the activity of the birds to which they were attached^56^.

**Table 4:** GPS tracking details for the six black sparrowhawk males on the Cape Peninsula over 2012-2015. We tagged three dark morph males and three white morph males with solar powered loggers. The table shows different breeding stages recorded for each individual. Individual Multiple Convex Polygons (MCP) and 10% Kernel Density Estimates (KDE) were calculated using GPS data from actively breeding individuals. We also report the breeding activity recorded for each individual and provide information on the dates of the breeding stages recorded and the stage which breeding reached for each breeding attempt.

Individual hawk	Morph	Date tagged	Total number of fixes obtained	*Breeding activity record	Number of fixes actively breeding	MCP size (km^2^)	Core territory size (10% KDE) (km^2^)	Total days tagged actively breeding
Zonnestraal	Dark	4 September 2012	23125	2012/10-Incubation; 2013/09- Nestlings; 2014/08-Prelay; 2015/08-Prelay	6739	16.29	4.61	240
Spillhaus	Dark	24 October 2014	21894	2014/12- Fledglings; 2015/11- Fledglings	4872	49.37	0.800	228
Tokai Arboretum	Dark	23 April 2013	15501	2013/04-Nestlings; 2014/05-Incubation; 2015/04-Prelay	4464	66.40	0.081	161
Stone Church	White	9 September 2013	17023	2013/10-Nestlings; 2014/07-Incubation	4686	25.88	0.062	177
Tokai Picnic	White	25 September 2014	6211	2014/10-Incubation; 2015/09- Fledglings	1928	59.43	1.23	64
Imhoff	White	17 August 2013	9388	2013/12- Fledglings; 2014/06-Prelay; 2015/11-Fledglings	5678	39.03	0.018	97

### Foraging activity and effort between morphs in relation to light levels

We examined morph ‘foraging activity’ in relation to weather condition, specifically light level. We defined individuals as actively foraging, when the birds were outside of their core nesting territory. To define this area, we used the core utilization distribution (UD) (i.e. 10% kernel density estimate (KDE)) of each GPS tagged individuals, estimated by means of a kernel density approach in R using the package ‘adehabitatHR’ classes and methods for home range estimation with the package “rgdal”. The KDE was derived by fitting contour lines (i.e. isopleths) based on the volume of the curve under the UD which defined the core polygon, whose area was then calculated (Table 4; Fieberg 2007). To estimate the 10% KDE for each individual, we only used GPS data from actively breeding individuals.

We assume that when birds were outside of their core nesting territories, they were actively foraging, although we recognise that they may have also been engaging other activities such as territorial defense, or avoiding begging females and chicks. For each individual in each hour, we then calculated the total number of fixes where the bird was actively foraging (i.e. outside of core nesting area) and not actively foraging (i.e. inside the core nesting area). This measure of foraging activity was then used as a two-vector response variable, fitted with a binomial distribution. This approach also therefore accounted for the slight variation in the number of total fixes received per hour. Data were analysed using Generalized Linear Mixed Models (GLMM), with individual bird fitted as a random factor to account for the lack of independence of the repeated hourly observations from the same tracked bird. Light condition experienced in each hour was fitted as an explanatory variable, together with morph type and the interaction between light levels and morph (Table 3). This was therefore a similar analytical approach as that taken by Tate *et al*. (2016) to examine for differential provisioning rates between morphs in relation to light levels. As foraging activity is likely to vary between breeding stages, (i.e. because food requirements vary between different stages of the breeding cycle), we also included ‘breeding stage’ as an additional explanatory variable, to control for potential differences in foraging activity between prelay, incubation, nestling and fledgling stages (Table 3).

We recognise that results may be influenced by differences in sample size (the number of fixes for each individual, i.e. we have a higher number of fixes for dark morphs; Table 4), as well as collection time and different sampling windows. We therefore re-ran our analysis – but randomly subsampled fixes from our dark birds to have a similar number of fixes as the white birds (dark morphs n = 12 300 fixes, white morphs, n = 12 292 fixes).

We also used a secondary measure of activity – ‘foraging effort’. For this we calculated foraging distance, which was the average hourly distance travelled. Although this measure is likely to be correlated with our other foraging measure (foraging activity), we thought it sensible to additionally include this variable, since any similar relationships with light levels would provide reassurance that our analyses were indeed capturing foraging behavior and not simply avoidance of the core nesting area (i.e. when males avoid begging females and young. This was observed frequently in our population, particularly when chicks were more developed (late nestling and fledgling stages), with females, and sometimes older offspring, displaying aggressive behaviour when males arrived on a territory, with or without food). Greater distances travelled outside of the core nesting area would thus confirm that the birds are indeed foraging or active, although we acknowledge they could still have been engaging in other activities besides foraging, such as territorial defense.

The distance travelled by breeding adults has been invoked as a good measure of foraging effort in a variety of bird species^57, 58^ including Verreaux’s Eagles^59^. During the active breeding period, we calculated the distance between each GPS fix using the package ‘geosphere’ in R. This was used to calculate the average hourly distance travelled by each individual, hereafter referred to as foraging effort. This foraging effort was used as the response variable and was analyzed in relation to light levels using the identical approach as for foraging activity, except that GLMMs were fitted using a Gaussian distribution (Table 3).

Whilst our main interests in these analyses were the relationship with light levels, we recognised this variable would be closely correlated with other weather variables such as rainfall, temperature and time of day. Thus, any relationship between light levels and foraging activity or effort could simply be due to a relationship with these other terms rather than light levels themselves. We therefore also included an additional analysis using the same model as described above, but substituted average hourly rainfall, average hourly temperature or time of day (and the quadratic term), and their interaction with morph (Table 3), to explore whether these other variables provided a better fit to the data than light levels. We then compared these models using their corrected Akaike Information Criterion scores (AICc), which is the AIC corrected for a small sample size, with the AICc scores for light level analyses (Tables 1 and 2).

### Habitat preference between the morphs

To explore habitat selection, we compared fixes of each bird with a series of random pseudo-absence points (i.e. points from the area that could have been visited by the observed individuals, but were apparently not) generated within each individuals home range from all fixes gathered during the breeding season^56^. Thus random fixes were generated within the area of the Minimum Convex Polygon (MCP), an area which encompassed 100% of all GPS fixes obtained for each individual (Supplementary material Fig. 3). Each individual’s MCP was estimated in R using the package ‘adehabitatHR’ classes and methods for home range estimation v.0.4.10^60^ with the package ‘rgdal v.0.9-1^61^ to process the spatial data. Three times as many pseudo-absence data points were chosen as observed points in the presented model^62, 63^. For our habitat preference analysis, to ensure habitat use was not just a reflection of the habitat around the nest site, we only used data outside of their core nesting areas (i.e. outside of the 10% KDE)(64) for both our bird GPS fixes and our random points.

In our analyses, we used the presences (GPS fixes = 1) or pseudo-absences (random points = 0) as a binary response variable, analysed within a GLMM with a binomial error structure. Explanatory variables in the model were habitat category (the six categories), male morph (dark or white) and the interaction between these two terms (which was our main variable of interest). Individuals were again treated as random effects to account for the lack of independence of points from the same bird.

Lastly, we ran a further habitat selection analysis exploring specifically if individuals showed stronger selection for denser tree canopy cover within their territories, depending on morph. For this analysis we again used a GLMM with the presences (GPS fixes = 1) or pseudo-absences (random points = 0) as a binary response variable, but with tree canopy closure (i.e. percentage of vegetation canopy cover), male morph (dark or white) and the interaction between these two terms as the explanatory variables. Individual was again treated as a random term in the model. To explicitly test the where significant differences in canopy cover selection occurred between morphs, we ran the same GLMM model using a moving subset of tree canopy cover^24^.

## Electronic supplementary material

Supplementary information
