# Supplementary material for: Morph specific foraging behavior by a polymorphic raptor under variable light conditions
Source: Sci Rep. 2017 Aug 22;7:9161. doi: 10.1038/s41598-017-07829-x (PMC5567351; doi:10.1038/s41598-017-07829-x)
Supplement: Supplementary file 1 — Supplementary information [file 41598_2017_7829_MOESM1_ESM.pdf]

Morph specific foraging behavior by a polymorphic raptor under variable light conditions.

Gareth John Tate* & Arjun Amar

Percy FitzPatrick Institute of African Ornithology, DST-NRF Centre of Excellence, University of Cape Town, ZA-7701 Rondebosch, South Africa

*Corresponding author: garethjtate@gmail.com

SUPPLEMENTARY MATERIAL

Table 1. Habitat landcover categories on the Cape Peninsula, South Africa (SANBI). We grouped our landcover into six logical categories for our habitat analysis and further classified these as open or closed habitat. Also shown are the percentages of GPS fixes for each SANBI landcover category.

SANBI landcover category	Grouped Category	Open/closed habitat	% of GPS fixes
indigenous forest	Closed habitat	Closed	0.4
thicket/dense bush	Closed habitat	Closed	8.2
cultivated comm fields (high)	Cultivated Land	Open	0
cultivated comm fields (med)	Cultivated Land	Open	<0.1
cultivated comm fields (low)	Cultivated Land	Open	0.03
cultivated comm pivots (high)	Cultivated Land	Open	0
cultivated comm pivots (med)	Cultivated Land	Open	0
cultivated comm pivots (low)	Cultivated Land	Open	0
cultivated orchards (high)	Cultivated Land	Open	0
cultivated orchards (med)	Cultivated Land	Open	0
cultivated orchards (low)	Cultivated Land	Open	0
cultivated vines (high)	Cultivated Land	Open	0.3
cultivated vines (med)	Cultivated Land	Open	0.7
cultivated vines (low)	Cultivated Land	Open	0.09
cultivated subsistence	Cultivated Land	Open	0
cultivated cane	Cultivated Land	Open	0
water seasonal	Open habitat	Open	<0.1
water permanent	Open habitat	Open	<0.1
wetlands	Open habitat	Open	0.2
woodland/open bush	Open habitat	Open	0.5
grassland	Open habitat	Open	2.0
shrubland fynbos	Open habitat	Open	4.7
low shrubland	Open habitat	Open	0.3

plantations / Woodlands clear-felled	Open habitat	Open	0
erosion (donga)	Open habitat	Open	0
bare none vegetated	Open habitat	Open	0.02
plantations / Woodlands mature	Plantations	Closed	34.1
plantations / Woodlands young	Plantations	Closed	3.4
urban informal (dense trees/bush)	Urban closed	Closed	0
urban residential (dense trees/bush)	Urban closed	Closed	15.3
urban smallholding (dense trees/bush)	Urban closed	Closed	0.3
urban sports and golf (dense trees/bush)	Urban closed	Closed	6.3
urban township (dense trees /bush)	Urban closed	Closed	0.04
urban village (dense trees/bush)	Urban closed	Closed	0
urban built up (dense trees /bush)	Urban closed	Closed	0
urban commercial	Urban open	Open	3.8
urban industrial	Urban open	Open	0.5
urban informal (open trees/bush)	Urban open	Open	<0.1
urban informal (low veg grass)	Urban open	Open	0
urban informal (bare)	Urban open	Open	<0.1
urban residential (open trees/bush)	Urban open	Open	0.09
urban residential (low veg/grass)	Urban open	Open	6.7
urban residential (bare)	Urban open	Open	0.2
urban school and sports ground	Urban open	Open	5.8
urban smallholding (open trees /bush)	Urban open	Open	0
urban smallholding (low veg/grass)	Urban open	Open	0.4
urban smallholding (bare)	Urban open	Open	<0.1
urban sports and golf (open tree/bush)	Urban open	Open	4.8
urban sports and golf (low veg/grass)	Urban open	Open	<0.1
urban sports and golf (bare)	Urban open	Open	0
urban township(open tree/bush)	Urban open	Open	0
urban township (low veg/grass)	Urban open	Open	0.5
urban township (bare)	Urban open	Open	0.02
urban village (open trees/bush)	Urban open	Open	0
urban village (low veg/ grass)	Urban open	Open	0
urban village (bare)	Urban open	Open	0
urban built up (open trees/bush)	Urban open	Open	0
urban built up (low veg/ grass)	Urban open	Open	0
urban built up (bare)	Urban open	Open	0.3

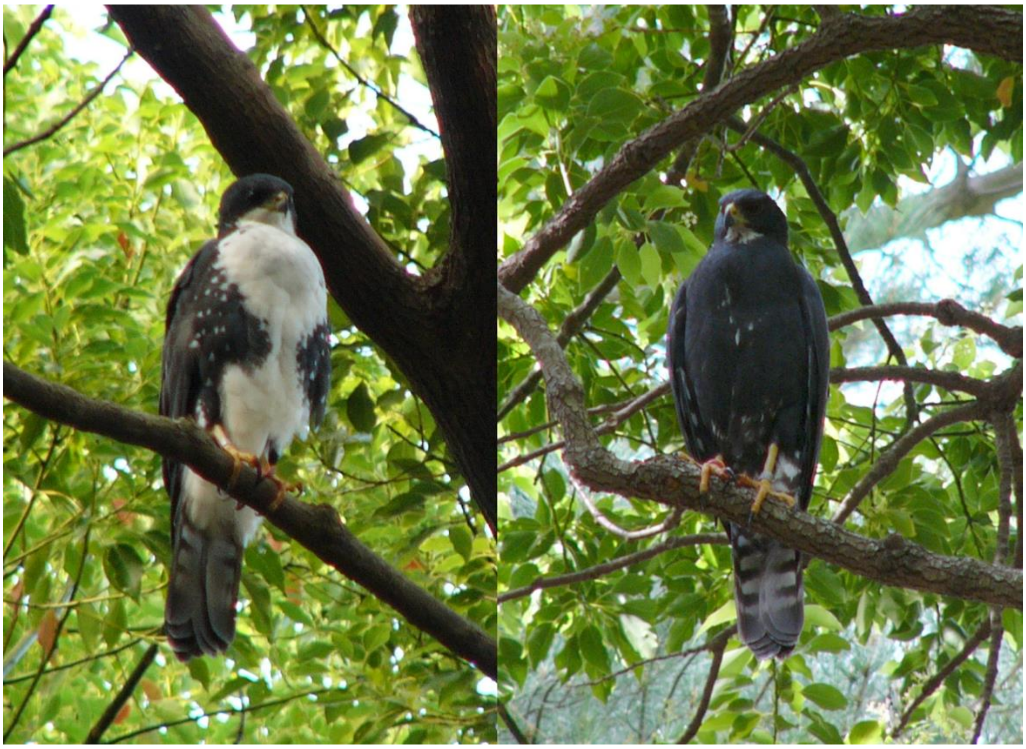

Figure 1. The black sparrowhawk *Accipiter melanoleucus* displays discrete polymorphism with adults occurring either as white (left) or dark (right) morphs. Photographs: Ann Koeslag.

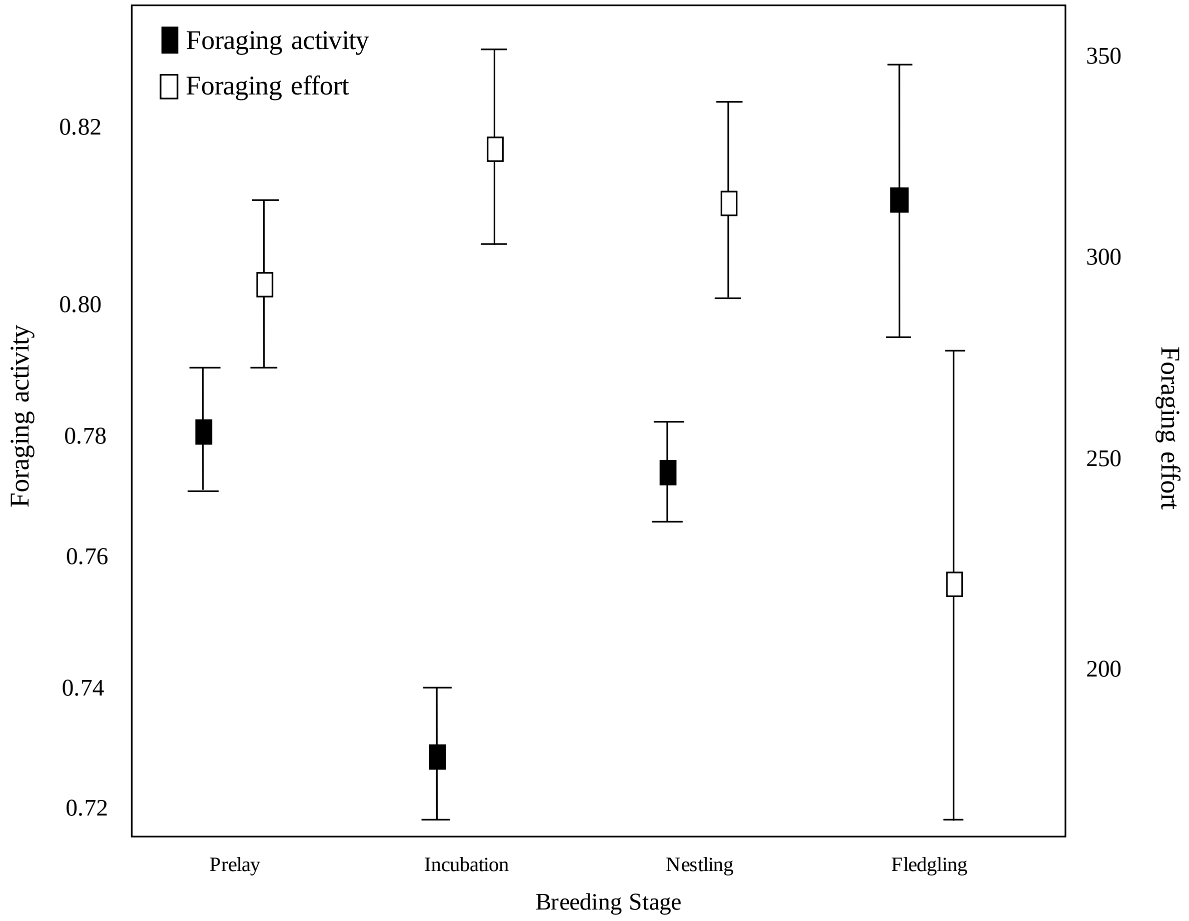

Figure 2. Black sparrowhawk foraging activity (measured as the probability of individuals being away from their core nesting territories) and foraging effort (measured as the average distance travelled (m)) across the various breeding stages on the Cape Peninsula.

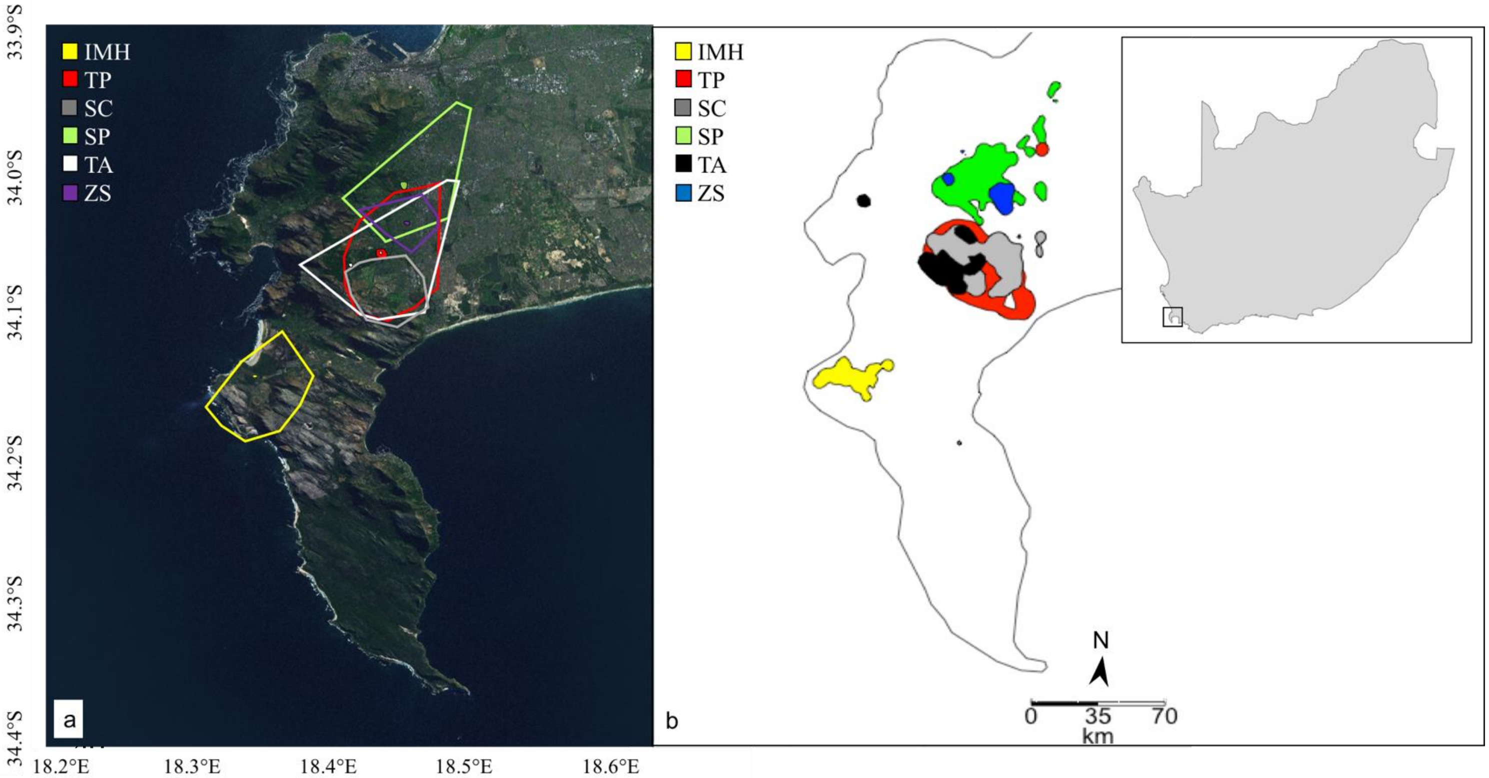

Figure 3a. Territory locations, Multiple Convex Polygons (MCP), territory cores (10%KDE) and **3b.** home ranges of our six GPS tagged male black sparrowhawks on the Cape Peninsula, South Africa from 2012-2015. Satellite image: ©2015 Google, DigitalGlobe. Maps generated in R, version 3.2.2 © https://www.r-project.org/ (R Development Core Team 2015).

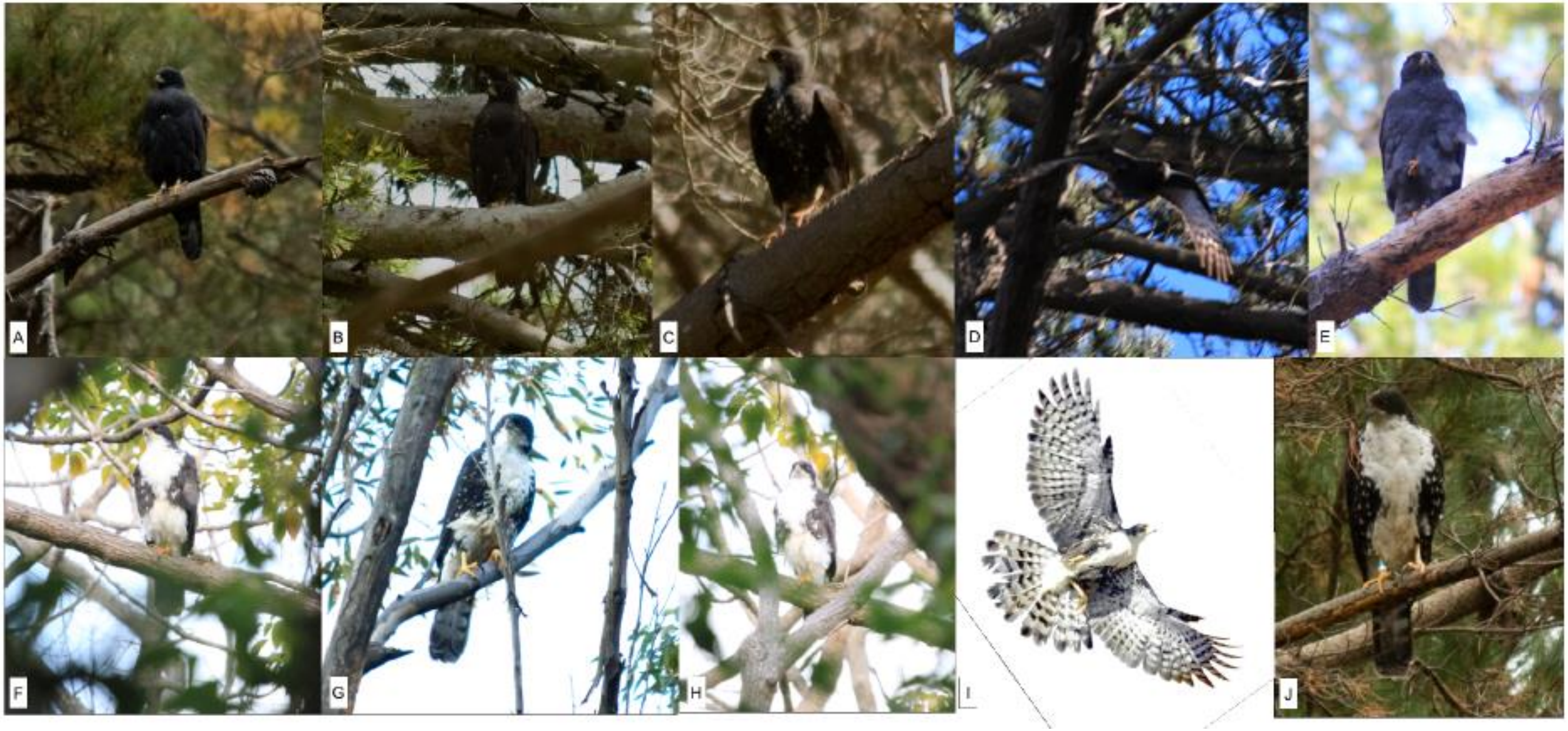

Figure 4. The following photographs illustrate the role of ambient light condition, habitat choice and background matching in concealing the different black sparrowhawk morphs from their prey. The top row (A-D) demonstrates how dense background cover and low-lit habitat facilitates crypsis in the dark morph while perched and when in flight (D). The image on the top right (E) shows how the uniform ventral plumage colour of the dark morph stands out against a bright, variable background and are thus more easily detected. The bottom row (F-I) shows how the light dappled ventral plumage of the white morph is concealed against bright open habitat background both while perched and when in flight (I). The image on the bottom right (J) shows how white plumage stands out against a darker background characterised by denser cover. (Photographs A-C, G & J-Ann Koeslag, D, E, F, H, I-Gareth Tate)
